# Chloroplast Genome Variation and Phylogenetic Relationships of Autochthonous Varieties of *Vitis vinifera* from the Don Valley

**DOI:** 10.3390/ijms25189928

**Published:** 2024-09-14

**Authors:** F. S. Sharko, K. O. Petrova, M. V. Patrushev, D. Y. Fedosov, S. V. Toshchakov

**Affiliations:** National Research Center “Kurchatov Institute”, Academician Kurchatov pl., 1, Moscow 123182, Russia; petrova_ko@nrcki.ru (K.O.P.); me@maxpatrushev.ru (M.V.P.); dimfedosov@yandex.ru (D.Y.F.); stepan.toshchakov@gmail.com (S.V.T.)

**Keywords:** *Vitis vinifera*, autochthonous varieties, assembly, annotation genome, de novo, chloroplast genome, phylogenetic analysis

## Abstract

The autochthonous grape varieties of the Don Valley, situated in southern Russia, constitute a distinctive element of regional cultural heritage. These varieties have been adapted over centuries to the region’s specific local climatic and soil conditions. For the most part, these varieties are not imported from other countries. They are closely related to varieties found in Crimea and the North Caucasus. In this study, we obtained the first complete, unfragmented sequences of the chloroplast genomes of eight autochthonous varieties from the Don Valley and one from Crimea. We also performed a comparative analysis of their genomic features. The size of *Vitis vinifera* chloroplast genome sequences varied from 160,925 to 160,991 bp, depending on the cultivar, with a uniform GC ratio of 37.38%. Each genome consists of four subregions: a single copy region (LSC) ranging from 89,158 to 89,336 bp, a small single copy region (SSC) ranging from 19,070 to 19,073 bp, and a pair of inverted repeat regions (IRa and IRb) in the range of 26,292 to 26,353 bp. The chloroplast genomes of the studied *V. vinifera* varieties contained 130 genes, including 85 protein-coding genes, 8 rRNA genes, and 37 tRNA genes. The sequence divergence analysis has enabled the identification of four highly variable regions, which may be utilized as potential markers for phylogenetic analysis. The analysis revealed the presence of 58 to 61 SSRs and multiple long repeated sequences in the chloroplast genomes of these varieties. The phylogenetic analyses of the sequences obtained and complete chloroplast genomes available from public databases indicated that the majority of autochthonous *V. vinifera* varieties do not have a direct origin from any European variety.

## 1. Introduction

The grape varieties cultivated in the Don Valley region of southern Russia constitute a distinctive gene pool. For the most part, they are not imported from other countries of the world, being close relatives of varieties found in Crimea and the Northern Caucasus [1]. These varieties were historically distributed across numerous villages situated along the right bank of the Don River. At the beginning of the 20th century, over 13,000 hectares of small family vineyards were located between Tsimlyansk and Aksay, spanning a distance of 200 km. Agricultural reforms during the Soviet era have led to the decline of Cossack family winemaking traditions. The varietal composition of the vineyards has also changed: preference was given to hybrid varieties [2], which were obtained by crossing *Vitis vinifera* with *Vitis amurensis* and American species.

Consequently, of the 42 varieties of the Don Valley described in previous publications [3,4], only six are widely represented. The most popular variety is Krasnostop Zolotovskiy. The rest can be found in private and public collections in southern Russia [1]. The genomic studies of indigenous varieties of the Don Valley, which have existed in this territory for centuries, can serve as a scientific basis to revive the interest of winemakers. The varieties in question are drought- and frost-resistant, and are well-suited to the local climate.

Krasnostop Zolotovskiy is a mid-ripening grape variety from the Black Sea basin [5]. It has small, three-lobed leaves and hermaphroditic flowers. The conical clusters are small and of medium density with dark blue, juicy berries. The ripening period in the Don Valley is 135 days, requiring a Huglin index (HI) value of 2800. It is resistant to fungal diseases and is used for high-quality red wines.

Krasnostop AZOS is an early-to-mid-ripening, interspecific hybrid derived from Krasnostop Zolotovskiy [6]. Its medium-sized, five-lobed leaves are dark green on top and slightly bristly underneath. With 135 days of growth and a 2600 HI, it shows phylloxera tolerance and is known for its juicy, dense-skinned berries.

Kokur Belyi, originating from Crimea [7] and introduced to the Don Valley before 1800, has large, deeply dissected leaves and produces medium-to-large conical clusters. It is a late-ripening variety, taking 160–170 days with 3300–3400 HI used for high-quality white, orange, sparkling, and fortified wines.

Dolgiy Skorospelyi is a separate variety, believed to be a clone of Kokur Belyi, with robust growth and medium-to-large cylindrical clusters. It has a 135–145-day ripening period but is sensitive to frost, fungal diseases, and phylloxera. Grapes are used for fresh consumption and wine production.

Plechistik is an ancestor of several varieties, including Krasnostop Zolotovskiy. It has medium-sized, strongly dissected leaves and functional female flowers. The medium cylindrical clusters produce small-to-medium berries with thin skin, requiring 135–140 days and 2800–2900 HI for ripening. It needs a pollinator like Tsimlyanskiy Chernyi to produce still and sparkling red wines.

Varyushkin is a Black Sea basin grape variety [8] with medium-sized, deeply dissected leaves and hermaphroditic flowers. It has conical clusters of medium density and rounded, black berries with thick skin and juicy flesh. The ripening period is 140–150 days, requiring about 3000 HI. It is frost-resistant and relatively resistant to mildew.

Sibirkovyi likely emerged in the 19th century from a cross between the local Bulanyi variety and the Romanian Pukhlyakovskiy Belyi (Coarne Alba) [9]. It has large, deeply dissected, five-lobed leaves and hermaphroditic flowers. The medium, slightly conical clusters produce oval, greenish-white berries with thin skin and juicy, melting flesh. The ripening period is about 130 days with 2650–2700 °C of active temperatures. This variety is prone to fungal diseases and frost, and is used for light still and sparkling wines.

Puklyakovskiy Belyi, a local name for Coarne Alba imported from the Balkans by a Cossack Pukhlyakov in the 19th century, has large, five-lobed, bubbly leaves. It features functionally female flowers and medium-sized cylindrical clusters with large, oval-ovate, greenish-white berries and thick skin. Its ripening period is about 140–145 days, requiring 3000–3200 HI, and its grapes are used fresh or for winemaking.

Kumshatskiy Belyi is related to Krasnostop Zolotovskiy and Plechistik and originates from the Tsimlyansk sub-region of the Don Valley [10]. It has large, deeply dissected, five-lobed leaves with a stretched middle blade, hermaphroditic flowers, and long, thick clusters. The medium-sized, round, greenish-white berries have fragile skin and juicy flesh. This medium–late-ripening variety takes about 180 days and requires 3300–3700 HI, producing aromatic, well-structured wine.

Chloroplasts are the primary organelles responsible for photosynthesis, the process through which plants transform solar energy into chemical energy [11]. The genomes of chloroplasts encode numerous key proteins that play a vital role in this photosynthetic process, such as enzymes and various accessory components [12]. In angiosperms, chloroplast DNA is usually inherited from the mother, whereas in gymnosperms, it is primarily passed down from the father [13]. This distinction makes chloroplast DNA a crucial focus for exploring heredity and genetic variation within plant populations [14]. Research on chloroplast genomes enables us to track evolutionary changes, to examine hybridization events between species, and to analyze the relationships among various plant varieties and species [15]. Chloroplast genomes comprise genes that play a role in the synthesis of a variety of metabolites [16], such as pigments, vitamins, and other essential compounds [12]. These substances are crucial for numerous cellular processes and significantly influence the color and flavor of plants.

The first sequenced chloroplast genome was that of *Marchantia polymorpha*, which occurred in 1986 [17]. Subsequently, numerous additional chloroplast genome sequences have been obtained. However, not all of these represented a complete sequence, and thus were only suitable for phylogenetic analysis with known limitations. Notwithstanding, specific regions of chloroplast genomes, including the maturase K (*matK*), ribulose bisphosphate carboxylase (*rbcL*), and yeast cadmium factor (*ycfL*) genes, have been extensively employed for DNA barcoding (species identification) [18]. However, these relatively short chloroplast markers were not suitable for distinguishing and analyzing the origin of cultivars. Their close relationship required a higher resolution. More extended sequences, specifically complete chloroplast genomes, may be suitable for full phylogenetic analysis. As sequencing technology continues to advance, methods for chloroplast genome sequencing have become more streamlined. Now, the analysis of an increasing number of chloroplast genome sequences has become more accessible [19,20]. The sequencing of complete chloroplast genomes was initially employed for the identification of cocoa cultivars [21]. Further applications of this approach permitted us to analyze the origin of varieties of significant crops, including hemp, date palm, tomato, rice, and other cultivars [22,23,24,25].

In this study, we present a phylogenetic analysis and comparative analysis of genomic features of complete, unfragmented sequences of the chloroplast genomes of eight autochthonous grape varieties from the Don Valley and one from Crimea. These sequences may be used in various fields, including molecular breeding, phylogenetics, and the conservation of endangered species [19,26,27].

## 2. Results

### 2.1. Subsection Characterization of the CP Genome Structure of Vitis vinifera Variety

**Figure 1:**
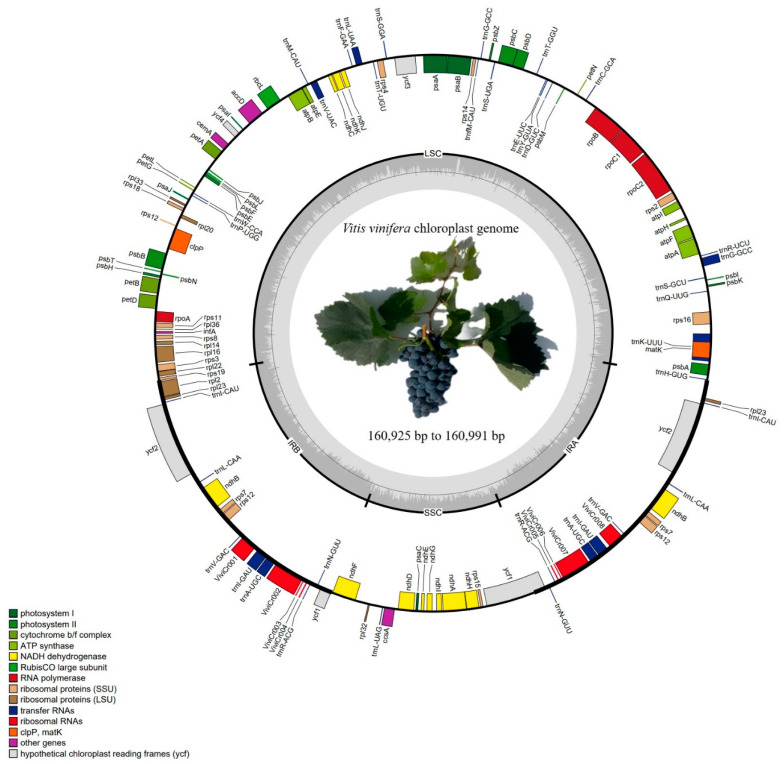
A circular chloroplast genome map of *V. vinifera* displaying genes located outside the circle that are transcribed in the clockwise direction, while those positioned inside the circle are transcribed counterclockwise. Genes are color-coded according to their functional groups. The dark gray region in the inner circle represents the GC content.

The length of the *V. vinifera* chloroplast genome sequences exhibited a range of 66 bp with a variation of 160,925 bp to 160,991 bp among different samples. Each chloroplast genome exhibited the characteristic quadripartite structure. It featured a large single copy (LSC) region spanning from 89,158 to 89,336 bp, a small single copy (SSC) region ranging between 19,070 and 19,073 bp, and a pair of inverted repeat (IR) regions (IRa and IRb) spanning from 26,292 to 26,353 bp. The average GC content across the entire chloroplast genome was 37.4%, with specific percentages of 43% in the IR regions, 35.33% in the LSC region, and 31.67% in the SSC region. No significant differences were observed between the ten *V. vinifera* chloroplast genomes. Additionally, all chloroplast genomes shared highly similar gene contents, encompassing a total of 130 genes. These included 85 protein-coding genes, 37 tRNA genes, and 8 rRNA genes (Figure 1). Among these, 16 genes were found to be duplicated, comprising 5 protein-coding genes (*ndhB*, *rpl2*, *rpl23*, *rps7*, and *ycf2*), 7 tRNA genes (*trnA-UGC*, *trnI-CAU*, *trnI-GAU*, *trnL-CAA*, *trnN-GUU*, *trnR-ACG*, and *trnV-GAC*), and 4 rRNA genes (*rrn16*, *rrn23*, *rrn4.5*, and *rrn5*). Of these genes, 45 were associated with photosynthesis, while 74 were involved in various chloroplast transcription and translation activities. Furthermore, 11 protein-coding genes and 8 tRNA genes contained one intron each, whereas the *clpP1*, *pafI*, and *rps12B* genes were found to contain two introns each (Table 1 and Supplementary Table S1).

### 2.2. IR Boundary Analysis

**Figure 2:**
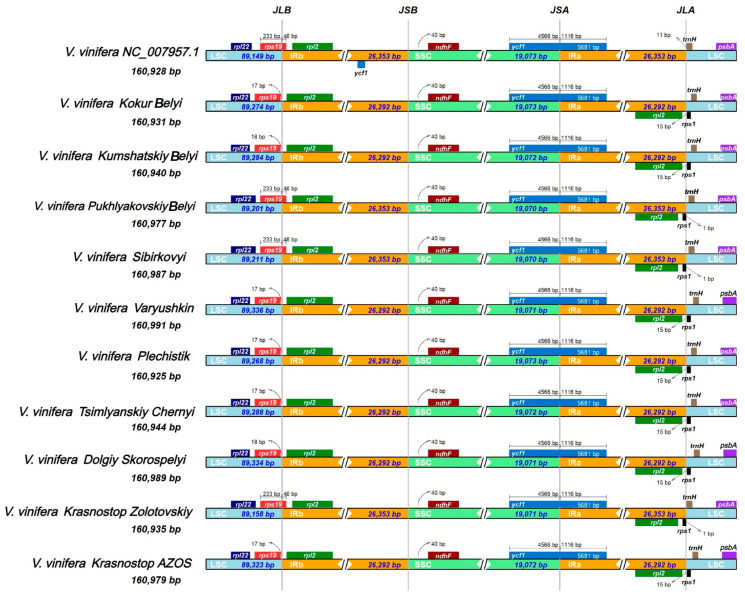
Comparison of the borders of the large single copy (LSC), small single copy (SSC), and inverted repeat (IR) regions in the chloroplast genomes of eleven *V. vinifera* varieties.

The genome structure of *V. vinifera*, including the number and order of genes, exhibited a high degree of conservation across different samples. Nevertheless, variations were observed, particularly at the boundaries between the large single copy (LSC), inverted repeat (IR), and small single copy (SSC) regions. Expansion or contraction of these boundaries within the chloroplast genome influences its overall size and is a key mechanism driving size differences among genomes [28]. To investigate these structural variations, we utilized the initial *V. vinifera* reference (NC_007957.1) as a control. Our analysis focused on comparing the boundaries of the IR, LSC, and SSC regions across different varieties. Figure 2 illustrates some of these variations; yet, upon closer inspection, significant differences were not observed in these boundaries. Notably, at the LSC/IRb boundary, a fragment of the *rps19* pseudogene was identified in one set of varieties, spanning 46 bp within the IRb region. In contrast, in another set, this gene was entirely contained within the LSC region, approximately 17–18 bp away from the IRb boundary. The *ycf1* gene was consistently present across all five chloroplast genomes at the SSC/IRa boundary, without variation. This region, SSC/IRa, was found to be the most conserved, with the boundary consistently located within the 5681 bp-long *ycf1* gene in all samples. Furthermore, the IRa/SSC boundary in all examined samples was found to span across the *trnH* and *rps1* genes, exhibiting minor positional shifts from one variety to another.

### 2.3. Genomic Sequence Divergence

**Figure 3:**
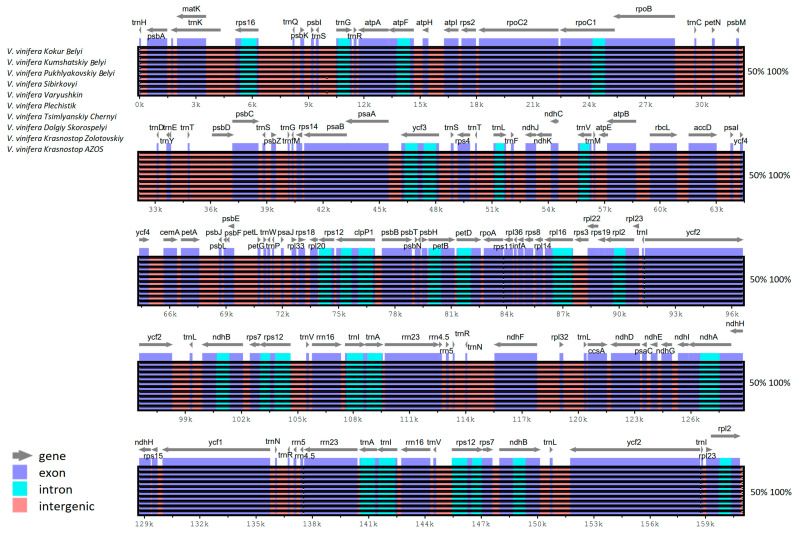
The sequence alignment of ten *V. vinifera* genomes in MVISTA is illustrated. The gray arrows above the alignment indicate the transcription directions of the genes. The y-axis displays the identity percentage, which ranges from 50% to 100% for each comparison. Genome regions are color-coded as exon, intron, and intergenic regions.

A sequence alignment analysis of the chloroplast genomes of the ten cultivars included in this study was performed using the chloroplast genome of *V. vinifera* (NC_007957.1) as a reference. The analysis demonstrated that the sequences were aligned in a consistent order and exhibited a high degree of conservation among the cultivars. We found that the non-coding regions showed greater divergence compared to protein-coding regions, indicating a more dynamic evolutionary history in these segments. Of the four major components of the chloroplast genome, the large single copy (LSC) region showed the most significant variability, whereas the inverted repeat (IR) region exhibited the least (Figure 3). This data suggests that the LSC region may be subject to evolutionary pressures that drive change, while the IR region remains stable and conserved.

**Figure 4:**
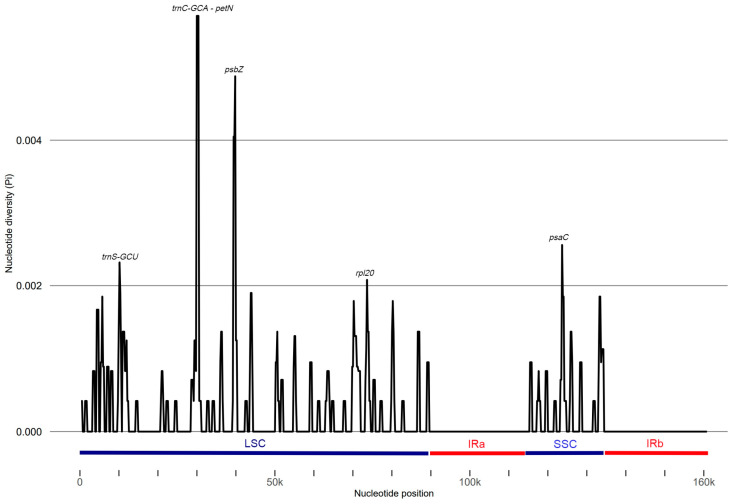
Nucleotide diversity (Pi) analysis for chloroplast genomes from the *V. vinifera* varieties with window length: 600 bp; step size: 200 bp.

Furthermore, we evaluated nucleotide diversity (Pi) values through the analysis of 600 bp segments of the chloroplast genome, which enabled us to identify specific regions of sequence divergence. The results indicated a range of Pi values from 0 to 0.0038 across the ten genomes studied, highlighting the extent of genetic variation present. Notably, we identified five regions with particularly high variability (Pi > 0.002): the *trnS(GCU)*, *trnC(GCA)-petN*, *psbZ*, and *rpl20* genes, all located within the LSC region, as well as the *psaC* gene situated in the small single copy (SSC) region (Figure 4). These findings point to specific loci that may be of interest for further investigations into the evolutionary dynamics and adaptation mechanisms of these cultivars. By focusing on these variable regions, future studies could enhance our understanding of the genetic diversity within chloroplast genomes and their implications for plant biology and breeding strategies.

### 2.4. Identification of Long Repeats

**Figure 5:**
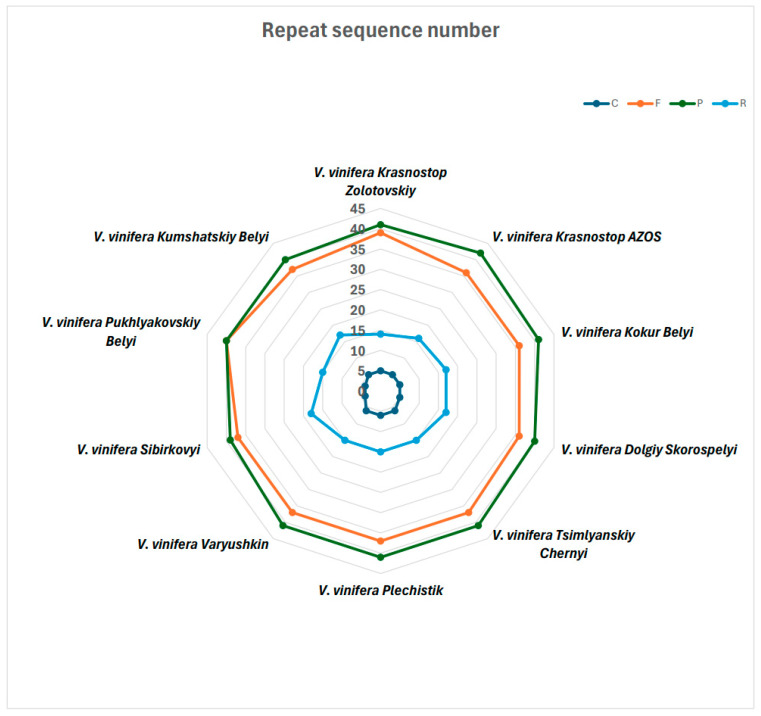
The number of long repeat sequences in the ten chloroplast genomes of *V. vinifera* (F: forward repeats; R: reverse repeats; P: palindromic repeats; C: complementary repeats).

Repetitive sequences are crucial for elucidating phylogenetic relationships among species and play a significant role in genome rearrangement [22]. In our study, we identified several types of repeats—forward (F), reverse (R), complementary (C), and palindromic (P)—in the chloroplast genomes of ten *V. vinifera* cultivars. In total, we detected 99 repeated sequences across these genomes, excepting the Sibirkovyi and Dolgiy Skorospelyi cultivars, which each contained 98 repeats (Figure 5 and Supplementary Table S2). The distribution of repeats exhibited considerable variation among the cultivars. The number of direct repeats ranged from 36 to 40, reverse repeats from 14 to 18, complementary repeats from 4 to 6, and palindromic repeats from 39 to 42. The Krasnostop AZOS variety exhibited the highest number of palindromic repeats at 42, while the counts of other types of repeat sequences were relatively consistent across the majority of *V. vinifera* cultivars. A significant finding of our analysis was that many of these repetitive sequences were predominantly located within non-coding regions, particularly in intergenic and intronic regions. This distribution suggests that these regions may play a critical role in the structural dynamics and regulatory mechanisms of the chloroplast genome [23]. Repetitive sequences in non-coding regions may offer insights into evolutionary processes, as these areas are more prone to variations that can drive genome rearrangement [24,25]. Further research on the functional implications of these repeats might illuminate their contributions to genetic diversity, adaptation, and potential breeding strategies in grapevine cultivars. Such investigations could enhance our understanding of the evolutionary significance of repetitive sequences in plant genomes as a whole.

### 2.5. Identification Simple Sequence Repeats

**Figure 6:**
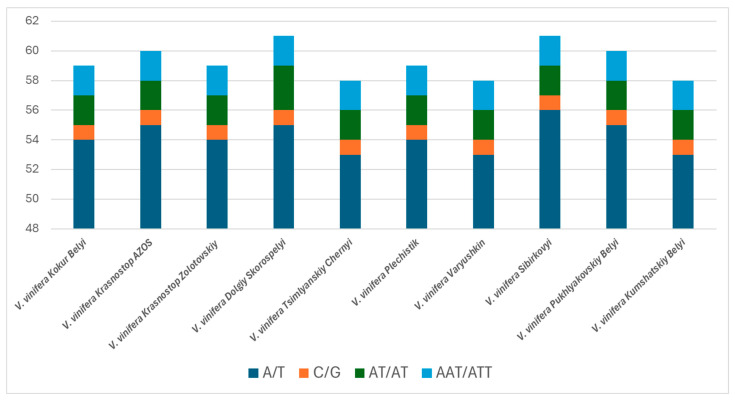
Number and types of SSRs detected in the ten chloroplast genomes of *V. vinifera*.

Simple sequence repeats (SSRs), commonly referred to as microsatellites, are characterized by relatively high mutation rates and copy number polymorphisms. This makes them invaluable as molecular markers for studies of genetic diversity, polymorphism, evolutionary dynamics, and plant breeding [29]. Furthermore, the study of complex SSRs can provide insights into the evolutionary processes that underpin microsatellite formation and variation [30]. In our study of the chloroplast genomes of grape varieties, we found that the SSR content varied across different cultivars. Specifically, the number of SSRs ranged from 58 in the varieties Kumshatskiy Belyi, Varyushkin, and Tsimlyanskiy Chernyi to 61 SSRs in the varieties Dolgiy Skorospelyi and Sibirkovyi. Among these, the percentage of mononucleotide repeats was highest in the Sibirkovyi variety, which exhibited 57 such repeats. In contrast, the number of dinucleotide and trinucleotide repeats was consistently recorded as two across most studied varieties, with the exception of the Dolgiy Skorospelyi variety, which had a third trinucleotide repeat (Figure 6 and Supplementary Table S3). The analysis revealed that the majority of mononucleotide and dinucleotide repeats were composedof A/T and AT/TA combinations, with other repeat types also predominantly featuring A and T as the major repeat units. In comparison, the occurrences of C and G were quite rare, indicating a bias in nucleotide composition for these SSRs. Upon examination of the chloroplast regions, it was observed that the highest SSR content was present in the large single copy (LSC) region, while the inverted repeat (IR) region exhibited the lowest SSR density. This distribution underscores the significance of the LSC region in contributing to genetic variability, which may have implications for understanding the adaptive potential and evolutionary pathway of grape varieties.

### 2.6. Selection Pressure Analysis

The ratios of nonsynonymous (Ka) to synonymous (Ks) substitutions for common protein-coding genes (PCGs) were calculated across ten grape genomes, with *V. vinifera* (NC_007957.1) and *V. vinifera subsp. sylvestris* (LC523806.1) as reference sequences (Supplementary Table S4). Out of the 85 common genes analyzed, we encountered challenges in determining the Ka/Ks ratios for many due to a lack of identical sequence variations or the absence of nonsynonymous or synonymous nucleotide substitutions. Additionally, in cases where calculations were possible, many did not yield significant results (*p*-value > 0.05). Interestingly, the majority of Ka/Ks ratios were found to be less than one, suggesting that most genes were under negative selection, indicating a preference for conserving protein function over time. However, one gene, *rpoC2*, exhibited potential signs of positive selection across all grape varieties, with a Ka/Ks ratio approaching 1. This could imply adaptive evolution in this gene, which may be associated with specific functions that confer advantages in certain environmental conditions or developmental processes [31,32].

### 2.7. Codon Usage Bias

We carried out an analysis of codon usage bias within the chloroplast genomes of *V. vinifera*, focusing on 85 protein-coding genes in order to gain a deeper understanding of the patterns in amino acid representation. Codons with a relative synonymous codon usage (RSCU) value greater than 1 were identified as being preferentially utilized for encoding specific amino acids, providing insights into genomic codon selection preferences [33]. This study examined the relative usage rates of codons across all protein-coding genes in the chloroplast genomes of ten distinct *V. vinifera* varieties, calculating the synonymous codon usage rates for comparison. The analysis revealed that all varieties contained a total of 61 different codons utilized to encode 22 amino acids (Supplementary Table S5). Interestingly, seven varieties shared the same total number of codons (23,273), while the varieties Tsimlyanskiy Chernyi and Plechistik had slightly fewer at 22,597, and the Sibirkovyi variety exhibited the smallest number of codons, totaling 23,184. Among the amino acids analyzed, leucine (*Leu*) emerged as the most frequently represented, accounting for 10.31–10.41% of codons, while cysteine (*Cys*) had the lowest representation at 1.11–1.13%. The AUU codon, which encodes isoleucine (*Ile*), was found to be the most common overall, occurring between 951 and 980 times across the genomes. Conversely, the UGC codon for cysteine (*Cys*) was the least prevalent, with only 71 to 74 total occurrences. In total, we identified 31 codon usage preferences (RSCU > 1) across the chloroplast genomes of all ten *V. vinifera* varieties, indicating significant bias in codon selection. The codons showcasing the highest and lowest RSCU values were UUA, encoding leucine, and GGC, which encodes glycine.

### 2.8. Phylogenetic Analysis

**Figure 7:**
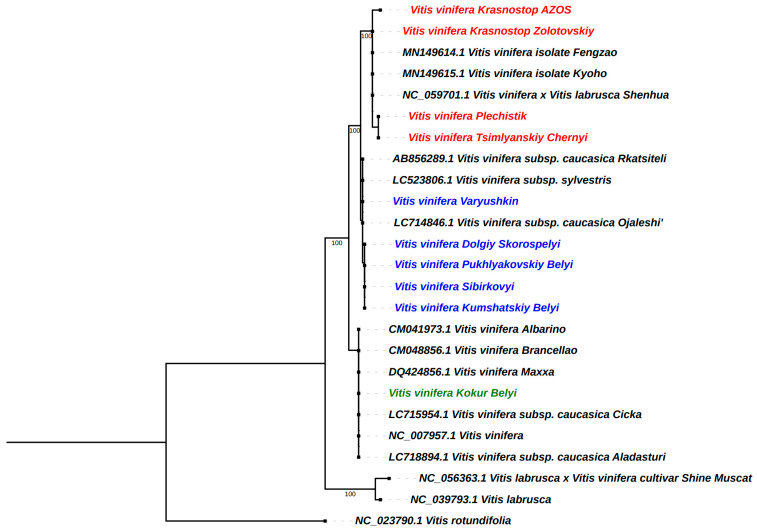
Phylogenetic reconstruction of *Vitis* using maximum likelihood (ML) methods based on PCGs. The values above the nodes are bootstrap support (BS) values.

To determine the phylogenetic position of autochthonous *V. vinifera* cultivars from the Don Valley, an ML (maximum likelihood) tree was constructed using the protein-coding genes of the *Vitis* chloroplast genomes obtained in the present study, as well as the other 15 members of the *Vitis* family downloaded from the NCBI database with *Vitis rotundifolia* as an outgroup. Phylogenetic analysis showed that, in addition to the variety *V. vinifera* Krasnostop Zolotovskiy, the varieties Tsimlyanskiy Chernyi, Plechistik, and Krasnostop AZOS also did not have direct genetic relationships with other Caucasian and European varieties of *V. vinifera* (Figure 7), as was shown earlier [34]. Instead, they formed a separate cluster with other autochthonous varieties in the genetic dendrogram of *V. vinifera*, which sharply contradicts earlier theories that suggested they originated from Eastern European varieties introduced into Russia. At the same time, the European and Caucasian varieties formed two separate groups with equally strong support (BS = 100). The Caucasian group included the majority of the autochthonous varieties analyzed in this study (Varyushkin, Dolgiy Skorospelyi, Puklyakovskiy Belyi, Sibirkovyi, and Kumshatskiy Belyi), while the European cluster included only one variety—Kokur Belyi. The aforementioned results demonstrate that chloroplast genome sequence analysis can offer valuable insights into the genetic background of *V. vinifera* varieties [35]. These can be used to aid in breeding and provide a molecular biological basis for cultivar identification.

## 3. Discussion

The present study involved the sequencing, assembly, and annotation of eight complete chloroplast genomes of autochthonous grape varieties from the Don Valley and one from Crimea. In order to gain a comprehensive understanding of the chloroplast genomes of grape varieties, we conducted a detailed comparison of the Krasnostop Zolotovskiy genome, which was previously analyzed in our laboratory [34], with those of other varieties. Our analysis encompassed the content of genes, long repeats, microsatellites, and stop codons. Additionally, we examined regions exhibiting the highest degree of variability and searched for genes displaying evidence of positive selection. Nevertheless, a comparison of chloroplast genomes in different cultivars of the same species revealed a high degree of conservation.

The conservation of chloroplast genomes in different grape varieties (*V. vinifera*) is of significant importance to our understanding of their genetic structures and evolutionary relationships [13]. The chloroplast genomes of grapes, like those of most higher plants [36], demonstrate a significant degree of homology between varieties, which allows them to be used for the study of phylogenetic relationships and the identification of varieties [37]. In this study, we aimed to clarify the phylogenetic relationships of autochthonous *V. vinifera* cultivars from the Don Valley by constructing a maximum likelihood (ML) tree based on protein-coding genes from *Vitis* chloroplast genomes. Our analysis showed that some local cultivars, including Tsimlyanskiy Chernyi, Plechistik, and Krasnostop AZOS, along with Krasnostop Zolotovskiy, do not have direct genetic relationships with other Caucasian and European varieties of *V. vinifera*. This finding contradicts the prevailing theory that these varieties originated from Eastern European varieties introduced to Russia [35]. The formation of a discrete cluster of these autochthonous varieties indicates the potential for disparate evolutionary trajectories and adaptation processes due to the distinctive ecological and climatic conditions of the Don Valley region [1]. This discrepancy emphasizes the significance of local cultivation practices and the particular ecological niches that these varieties occupy, thereby enabling them to evolve distinctive genetic attributes over time. The results also demonstrate a clear separation between the European and Caucasian groups within the genetic dendrogram. The Caucasian cluster is represented mainly by the varieties studied in the work, while the European cluster is represented by one variety, Kokur Belyi. This difference raises questions about the historical and geographic factors that contributed to the genetic diversity observed among *V. vinifera* cultivars. This may also challenge the assumption that many autochthonous varieties share a common origin with their European counterparts.

The grape chloroplast genome features a circular molecular structure containing dozens of genes responsible for photosynthesis and other metabolic functions. Throughout evolution, most genes related to essential chloroplast functions have remained largely unchanged. This indicates a strong selective pressure for their conservation [38]. Key genes, such as those encoding photosystem subunits and enzymes involved in chlorophyll synthesis, maintain conserved sequences, underscoring their crucial role in plant survival [39]. The results of this study emphasize the significance of simple sequence repeats (SSRs) in the genetic analysis of grapes, which may result in future studies using these markers for genetic mapping, cultivar identification, and the improvement of breeding programs [40]. Our results indicate marked differences in SSR content between different cultivars, with the number of SSRs varying from 58 to 61 among the cultivars analyzed. This variability underscores the potential of SSRs for use in cultivar discrimination and understanding the genetic structure of grape populations. Investigating the functional consequences of SSR variation in grape chloroplast genomes enhances our understanding of evolutionary processes. It also helps to determine the mechanisms of diversification of this economically important plant [41].

Additionally, our results revealed differences in codon usage among *V. vinifera* cultivars, illuminating their evolutionary adaptations [42]. Understanding these codon preferences is crucial for developing genetic markers for breeding and conservation, as well as for studying the functional roles of proteins in grapes.

Our research findings have significant implications for grapevine breeding and conservation strategies. The detailed genetic characterization of these varieties may result in the discovery of unique traits that are valuable for breeding programs focused on disease resistance, climate adaptability, and general agronomic performance [43]. By analyzing chloroplast genome sequences, researchers and breeders can gain a deeper understanding of the genetic basis of *V. vinifera*, leading to more informed selection of parental varieties and improved cultivar development.

## 4. Materials and Methods

### 4.1. Plant Materials, DNA Extraction, and Sequencing

In this study, ten samples of *Vitis vinifera* were collected from different places in southern Russia (Supplementary Table S1). Fresh and healthy leaves of these ten samples were collected. The DNA extraction protocol used in this research was adjusted from that of Sandra Lo Piccolo (2012) [44]. Modifications included performing all centrifugation procedures at 3500× *g* and 4 °C [8,45]. The centrifugation time was 15 min and 45 min for DNA extraction with chloroform-isoamyl alcohol and isopropanol precipitation of DNA, respectively. The concentration and quality of extracted DNA was assessed using a Nanodrop 1000 device (Thermo Fischer Scientific, Waltham, MA, USA) and a Qubit fluorometer with the Qubit™ dsDNA BR Assay Kit (Thermo Fischer Scientific, Waltham, MA, USA). DNA fragment libraries were prepared for Illumina sequencing using the NEBNext^®^ Ultra™ II DNA Library Prep Kit for Illumina^®^ (New England BioLabs, Ipswich, MA, USA) according to the manufacturer’s protocol. The resulting libraries were sequenced on the NovaSeq 6000 platform (Illumina, San Diego, CA, USA) using 2 × 150 bp paired-end chemistry.

### 4.2. Genome Assembly and Annotation

Fastp v0.23.4 [35] was employed to eliminate adapter-containing sequences and low-quality reads from the data. This process resulted in clean read yields ranging from 56.20 to 129.32 GB. The chloroplast genomes were then assembled using GetOrganelle software (version 1.7.7.0) [36] with default settings. Following assembly, the contig underwent correction with Pilon [37] and validation through short-read mapping using Bowtie2 (version 2.4.4) [38]. The annotation of the assembled chloroplast genomes was conducted using GeSeq v.1 software [39]. For the identification of tRNA genes, we utilized tRNAscanSE 2.0.12 [40] with default parameters. The rRNA components of the chloroplast genome were annotated using BLASTN software (version 2.12.0) [41]. The circular map of the chloroplast genome was visualized using OGDRAW 1.3.1 [42]. All read data generated during this study have been documented and deposited in the NCBI Sequence Read Archive (SRA) and can be accessed under the respective accession number PRJNA1011053.

### 4.3. Comparative Genome Analysis

The nine complete chloroplast genomes of *V. vinifera* in this study and another variety, *V. vinifera* Krasnostop Zolotovskiy, were compared using the MVISTA [46] program with the shuffle-LAGAN model [47], with *V. vinifera* (NC_007957.1) as the reference. The IRSCOPE program [48] was applied to analyze the LSC, IR, and LSC boundary locations in eleven complete *Vitis* chloroplast genomes. Sliding window analysis was conducted to determine the nucleotide variability (Pi) of the complete chloroplast genome using DnaSP v6 [49] after sequence alignment with MAFFT v7.427 [50]. The sliding window length was set as 600 bp, with a step size of 200 bp. In addition, DnaSP v6 was adopted to calculate insertions and deletions (InDels) and Pi for highly variable regions.

### 4.4. Analysis of the Cp Genome for Repetitive Sequences

Simple sequence repeats (SSRs) in the chloroplast genomes of ten *Vitis* varieties were analyzed using MISA v2.1 software [51]. The minimum repeat units were configured as follows: 10 for mononucleotide SSRs, 5 for dinucleotide SSRs, 4 for trinucleotide SSRs, and 3 for tetranucleotide, pentanucleotide, and hexanucleotide SSRs. Additionally, REPuter v1 software [52] was employed to identify different types of repeats, including forward (F), reverse (R), palindrome (P), and complementary (C), with criteria set for a minimum repeat size of 30 bp and a Hamming distance of 3.

### 4.5. Codon Preference Analysis

Codon W v1.4.4 software [53] was utilized for codon analysis and to determine relative synonymous codon usage (RSCU) values for comparative mapping. RSCU reflects the likelihood of employing synonymous codons to encode a particular amino acid: an RSCU value greater than 1 indicates that the codon is frequently used; an RSCU value equal to 1 suggests no bias in codon usage; and an RSCU value of less than 1 indicates that the codon is rarely used.

### 4.6. Selective Analysis

To evaluate selective pressure on the *Vitis* varieties, we analyzed the Ka/Ks ratio (where Ka represents the nonsynonymous substitution rate and Ks denotes the synonymous substitution rate) in a study involving ten grapevine varieties. Using PhyloSuite v1.2.3 [54], we extracted 85 common protein-coding genes (PCGs) and translated them into their corresponding amino acids. We then utilized KaKs_calculator 3.0 [55] with ParaAT v2.0 [56] to automatically generate intermediate files and compute the Ka/Ks values, discarding results with excessively high Na values, which were likely due to an extremely low synonymous substitution ratio. A Ka/Ks ratio greater than 1 indicated that the gene pair was under positive selection, while a ratio lower than 1 suggested purifying selection.

### 4.7. Phylogenetic Analysis

For the phylogenetic analysis, the complete chloroplast genomes of 15 samples within the *Vitis* family, available by June 2024, were downloaded from the National Center for Biotechnology Information (NCBI). *Vitis rotundifolia* were chosen as the outgroups. Common protein-coding genes (PCGs) were extracted and aligned using MAFFT v7.427 [50] using the iterative method (G-INS-i) using default parameter settings. To construct the maximum likelihood (ML) phylogenetic tree, the RAxML algorithm [57] was employed and bootstrap values were obtained from 1000 replicates. The resulting tree was visualized using the iTOL service [58].

## 5. Conclusions

For the first time, a comparative analysis of chloroplasts in grape genomes was carried out. But, since the chloroplast genomes in open databases today are quite small, we made a significant contribution to the further study of chloroplasts as markers for studying the adaptive and evolutionary mechanisms of this genus. Studying the chloroplast genomes of grapes can help biodiversity conservation programs by tracking genetic resources and risks to extinction in certain varieties. Thus, chloroplast genomes are an important object of scientific research due to their importance in photosynthesis, heredity, environmental adaptation, and biotechnology.

## Figures and Tables

**Table 1:** Annotated genes and their classification in the chloroplast genomes of *V. vinifera* varieties.

Category	Group	Genes
Photosynthesis	Photosystem I	*psaA*, *psaB*, *psaC*, *psaI*, *psaJ*
	Photosystem II	*psbA*, *psbB*, *psbC*, *psbD*, *psbE*, *psbF*, *psbH*, *psbI*, *psbJ*, *psbK*, *psbL*, *psbM*, *psbT*, *psbZ*, *psbN*
	ATP synthase	*atpA*, *atpB*, *atpE*, *atpF* *, *atpH*, *atpI*
	Cytochrome b/f complex	*petA*, *petB* *, *petD* *, *petN*, *petL*, *petG*
	Cytochrome C synthesis	*ccsA*
	Rubisco	*rbcL*
	NADPH dehydrogenase	*ndhA* *, *ndhB* (×2) *, *ndhC*, *ndhD*, *ndhE*, *ndhF*, *ndhG*, *ndhH*, *ndhI*, *ndhJ*, *ndhK*
Self-replication	RNA polymerase	*rpoA*, *rpoB*, *rpoC2*, *rpoC1* *
	Ribosomal proteins	*rps2* (×2), *rps3*, *rps4*, *rps7* (×2), *rps8*, *rps11*, *rps12* (×2) *, *rps14*, *rps15*, *rps16* *, *rps18*, *rps19*, *rpl2* *, *rpl14*, *rpl16* *, *rpl20*, *rpl22*, *rpl23* (×2), *rpl32*, *rpl33*, *rpl36*
	Translation initiation factor	*infA*
	Ribosomal RNA	*rrn16* (×2), *rrn23* (×2),
		*rrn4.5* (×2), *rrn5* (×2)
	Transfer RNA	*trna-UGC* (×2) *, *trnC-GCA*, *trnD-GUC*, *trnE-UUC*, *trnF-GAA*, *trnfM-CAU*, *trnG-GCC*, *trnG-UCC* *, *trnH-GUG*, *trnI-CAU* (×2), *trnI-GAU* * (×2), *trnK-UUU* *, *trnL-CAA* (×2), *trnL-UAA* *, *trnL-UAG*, *trnM-CAU*, *trnN-GUU* (×2), *trnP-UGG*, *trnQ-UUG*, *trnR-ACG* (×2), *trnR-UCU*, *trnS-GCU*, *trnS-GGA*, *trnS-UGA*, *trnT-GGU*, *trnT-UGU*, *trnV-GAC* (×2), *trnV-UAC* *, *trnW-CCA*, *trnY-GUA*
Other genes	Maturase	*matK*
	Envelope membrane protein	*cemA*
	Acetyl-CoA carboxylase	*accD*
	Proteolysis	*ClpP* **
	Conserved ORFs	*ycf1* (×2), *ycf2* (×2), *ycf3*, *ycf4*

*: Gene containing a single intron; **: Gene containing two introns; (×2): Number of genes with multiple copies.

## Data Availability

The chloroplast sequences of *V. vinifera* varieties were deposited into the National Center for Biotechnology Information (NCBI) repository, and the accession number is PRJNA1011053.

## Supplementary Materials

The following supporting information can be downloaded at: https://www.mdpi.com/article/10.3390/ijms25189928/s1.
